# Visual Dysfunction in Posterior Cortical Atrophy: A Masquerade

**DOI:** 10.7759/cureus.30621

**Published:** 2022-10-23

**Authors:** Aditya Mahindru, Ruthshee Suresh, Pradeep Patil

**Affiliations:** 1 Psychiatry and Behavioral Sciences, Jawaharlal Nehru Medical College, Datta Meghe Institute of Higher Education and Research, Wardha, IND; 2 Psychiatry, National Institute of Mental Health and Neurosciences, Bangalore, IND; 3 Psychiatry, Jawaharlal Nehru Medical College, Datta Meghe Institute of Higher Education and Research, Wardha, IND

**Keywords:** visuoperceptual, visuospatial, alzheimer's disease, dementia, posterior cortical atrophy

## Abstract

Posterior cortical atrophy, considered an atypical dementia, is a syndrome characterised by dysfunction of posterior cortical regions with prominent visuospatial and visuoperceptual impairment at presentation. We report the case of posterior cortical atrophy, which was diagnosed six years after the onset of visual symptoms. The patient is a 67-year-old married gentleman, with six years history of visual impairment, characterised by difficulty in locating and manipulating door handles, overreaching objects and difficulty in depth perception. He had a history of repeated visits to ophthalmologists and underwent multiple unsuccessful changes in eyeglasses and a cataract surgery to correct acuity. The patient also developed recent memory deficits about two years back, insidious in onset and gradually progressed and symptoms of visual and auditory hallucinations about six months back. Cognitive and functional assessments, and imaging findings were consistent with a diagnosis of posterior cortical atrophy, possible Alzheimer’s disease. The patient was started on cognitive enhancers and low dose antipsychotics. He was engaged in meaningful and cognitively stimulating activities. Environmental manipulations and home safety recommendations for visual impairment were conveyed to the family. In the early stages of posterior cortical atrophy, visual symptoms predominate, while episodic memory, executive functioning, language, and insight are substantially retained. Better identification, prognosis, and treatment of posterior cortical atrophy will result from increased knowledge and understanding of the condition among neurologists, psychiatrists, general doctors, ophthalmologists, and optometrists.

## Introduction

Posterior cortical atrophy (PCA), considered an atypical dementia, is a syndrome characterised by dysfunction of posterior cortical areas, with prominent visuospatial and visuoperceptual impairment at onset [1]. While PCA was formerly thought to be very uncommon, recent research reveals that it really plays a role in at least 13% of all occurrences of Alzheimer's disease (AD) in those younger than 65 years of age [2]. Due to the lack of knowledge of AD's no amnestic manifestations and the earlier age of onset, it is likely to be underrecognized and misdiagnosed. Here we report a case of PCA which was finally diagnosed after six years of onset of visual complaints.

## Case presentation

A 67-year-old married gentleman, retired engineer from a middle-class nuclear family, presented to us with agitation, physical aggression, and visual and auditory hallucinations that had first appeared roughly six months prior and had worsened since then. He had no known medical conditions or family history of neuropsychiatric illness. The patient received in-patient treatment to better understand his diagnosis and to better manage his condition. After a more in-depth conversation with the patient's spouse, it was discovered that the patient had begun having trouble opening doors around six years ago. When lighting candles or playing carrom, he had a tendency to overreach for the flame or the ball. He had difficulty reading the newspaper as well. He had been to many different ophthalmologists, gone through several pairs of glasses, and even had cataract surgery, all to no avail. His problems with eyesight and depth perception issues, however, worsened with time. He complained of trouble watching the television and eventually stopped doing so. He gradually developed difficulty in driving his car and would have difficulty in finding the way even in familiar places. The patient eventually stopped driving and going out of the house by himself. Even navigating to and using the restroom became a challenge for him. He would prefer urinating on the bathroom floor as he was unable to locate the commode. These symptoms gradually progressed in severity in the last five years. The patient's recent memory had deteriorated in the last two years, causing him to lose his keys, forget important discussions, and misplace items. The family had written off the memory issues as inevitable ageing and hence had not sought medical help. The patient also struggled to get dressed in the morning, as he had trouble buttoning his shirt and zipping up his trousers. He had difficulty performing various basic activities of daily living. The patient began experiencing auditory and visual hallucinations around six months ago. He was irritable and verbally abusive in response to the hallucinations. He reported that his siblings and neighbours were threatening to take away his property. He appeared to make conversation with people who are not visible or audible to others. He made aggressive gestures in response to the hallucinations and would get physically and verbally abusive towards his family members.

**Figure 1:**
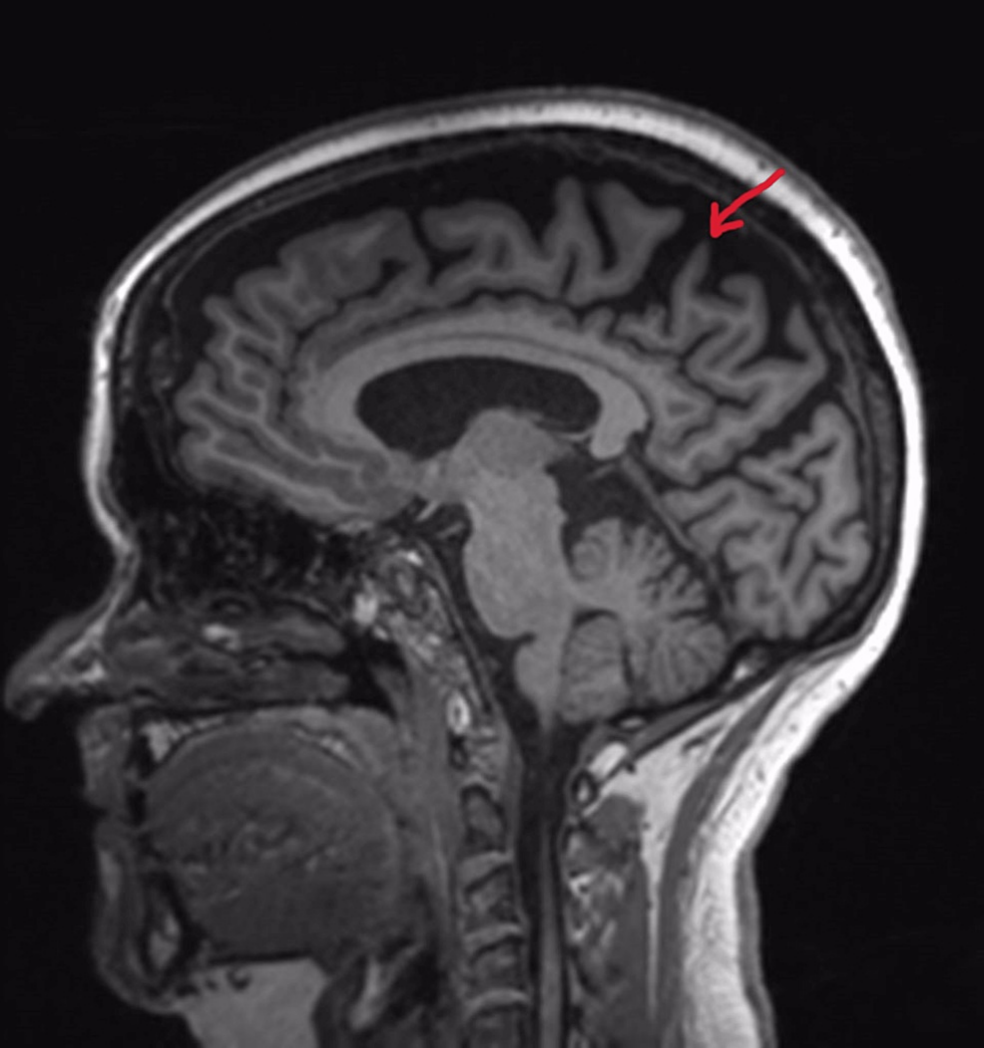
MRI Brain Sagittal T1 indicating generalised cortical atrophy with prominent atrophy in parietal and occipital regions

**Figure 2:**
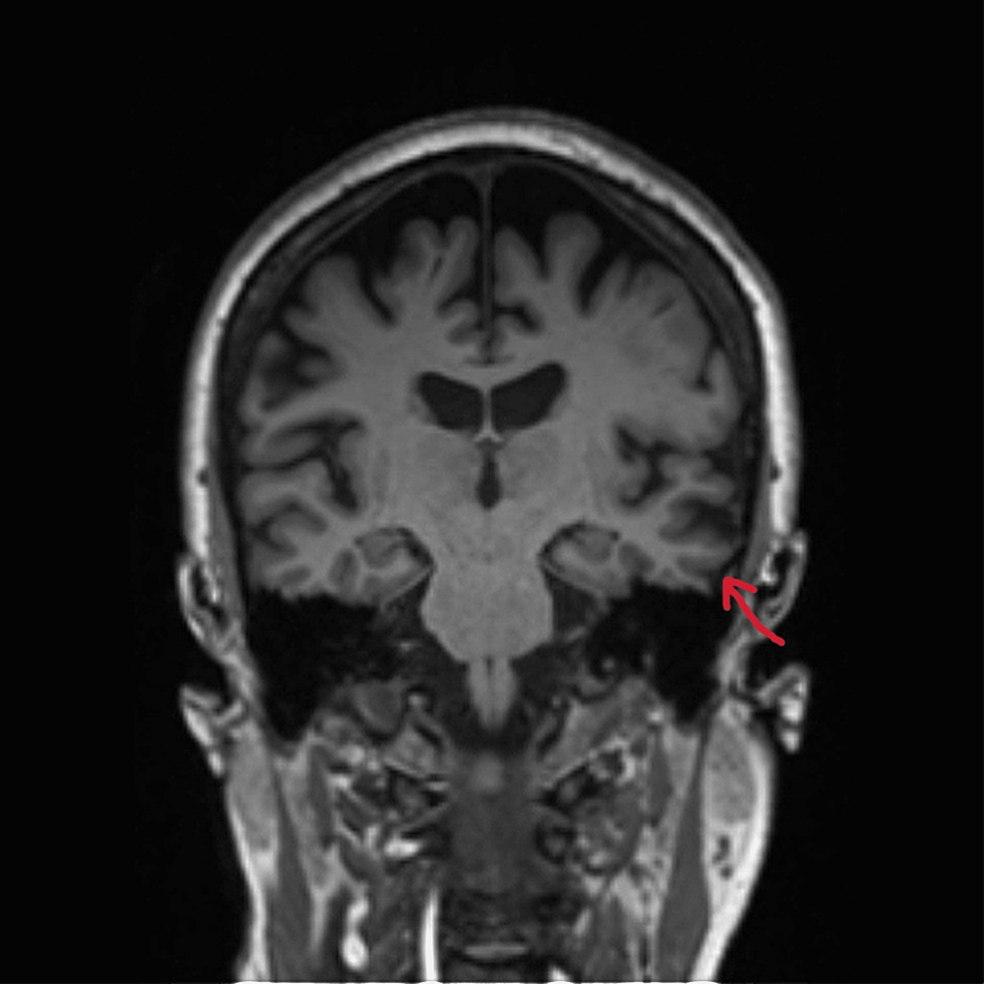
MRI Brain Coronal T1 indicating generalised cortical atrophy with prominent atrophy in parietal and occipital regions

**Figure 3:**
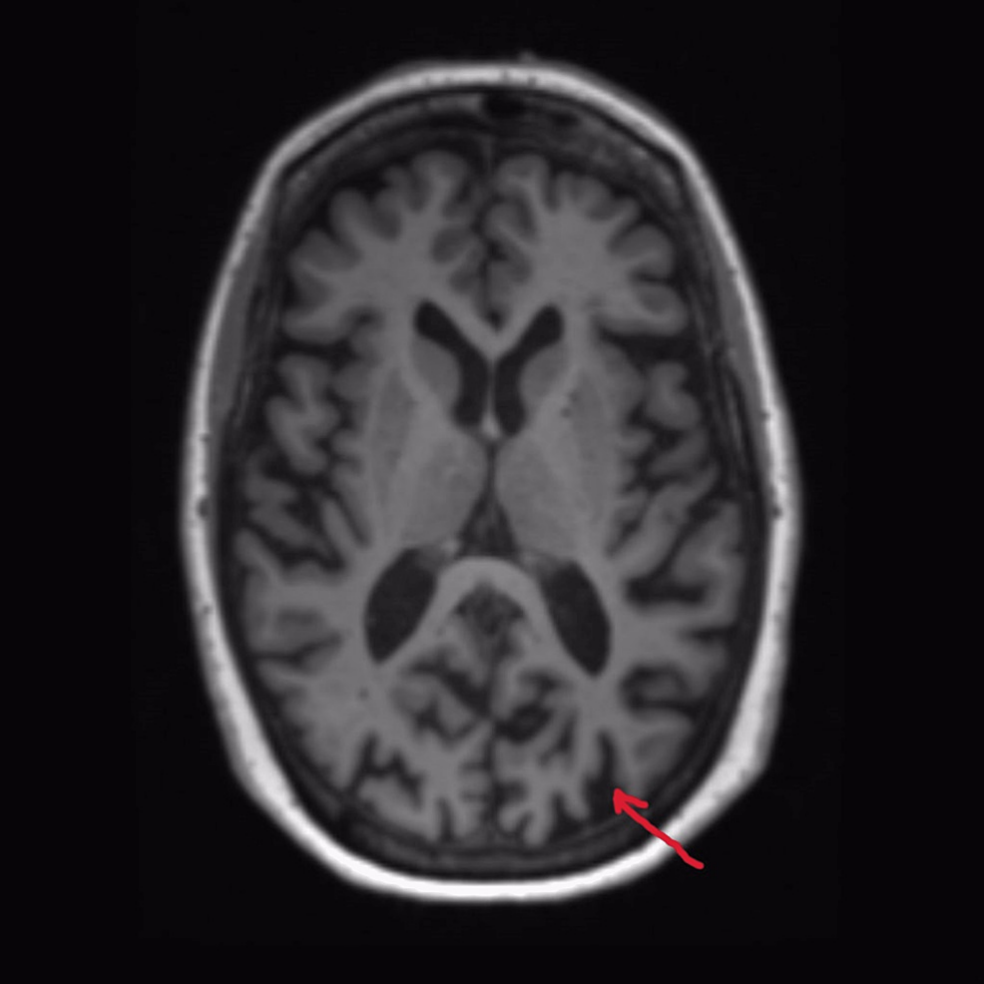
MRI Brain Axial T1 indicating generalised cortical atrophy with prominent atrophy in parietal and occipital regions

On mental status examination, the patient was agitated and uncooperative for an interview. His psychomotor activity was increased, and he was physically aggressive towards the treating team. De-escalation and chemical sedation were used to manage the agitation during the first three days of admission. Blood investigations revealed no abnormality in metabolic parameters and no signs of infection. General physical examination was normal and no signs of pain or constipation were noted. On neurological examination, no obvious abnormality was noted. He did not have any tremors, rigidity, bradykinesia or postural instability. On ophthalmic examination, visual acuity was 3/60 in each eye. Extraocular movements were normal. Depth of the anterior chamber was normal with no cells or flare. Pupils were round, regular and reactive. Both eyes had intraocular lens in situ. On fundoscopic examination, the optic disc was normal with good foveal reflex. Visual field testing was not done. MRI brain was done which showed generalised cortical atrophy with significant atrophy in the occipital and parietal lobes (Figure 1-3). 

He was also started on syrup risperidone 0.5ml two times a day which was later changed to tablet formulation and dose was increased to 0.5mg in the morning at 8 am and 1mg at 4 pm. The patient’s agitation and hallucinatory behaviour gradually reduced over the course of admission. Addenbrooke’s Cognitive Assessment was administered in which he scored 30/100 with impairment in attention (3/18), memory (4/26), fluency (4/14) and visuospatial (2/16). He scored relatively better in language (17/26). A diagnosis of posterior cortical atrophy, possible Alzheimer’s disease was considered. He was started on rivastigmine transdermal patch 4.6mg/24 hours. On serial mental status examinations he was noted to have a depressed mood and crying spells in view of which tablet escitalopram 5mg was started. To aid the patient’s visual impairments, his room and bathroom were labelled using boards and fonts of high contrasting colours. He was engaged in cognitively stimulating activities like listening to music and audiobooks and going for walks in the garden. He was noted to have an improvement in his affect. His social interactions and engagement with the treating team and nursing staff improved. No aggression or agitation was noted and he was discharged to go home. Environmental manipulations and home safety recommendations for visual impairment were conveyed to the family. 

## Discussion

Benson and co-workers [3] first used the term posterior cortical atrophy (PCA) to characterise a group of patients who had abnormalities in higher-order visual processing in addition to hallmarks of the Gerstmann and Balint syndromes but relatively preserved episodic memory. A neurodegenerative disorder, PCA typically affects the parietal and occipital lobes. It is characterized by early prominent visual dysfunction that cannot be explained by ocular causes. The vast majority of those diagnosed with posterior cortical atrophy present between the ages of 50 and 65. However, there are also descriptions of people whose symptoms didn't appear until their 90s [4,5]. The core features of PCA include visuospatial and perceptual deficits as well as features of Gerstmann syndrome (acalculia, left-right disorientation, finger agnosia, and agraphia), Balint syndrome (oculomotor apraxia, optic ataxia, and simultanagnosia), alexia, and apraxia [6,7]. Patients with PCA have been reported to have tried many pairs of corrective lenses or even surgical treatments to improve their vision, all to no avail. Due to their inability to judge distances accurately, some people can get into minor car accidents [8]. Difficulty in reading digital and analogue clocks is another common early feature. In our patient too, the onset was characterised by visuospatial and visuoperceptual abnormalities like manipulating door handles. He presumably had apraxia and issues with depth perception, which made everyday tasks like lighting candles, aiming for coins while playing carrom, urinating in the commode precisely, and driving a car difficult for him. He also eventually developed wayfinding difficulties outside and inside the home due to the visuospatial deficits. Like most patients of PCA, ours was first seen by an ophthalmologist who tried prescribing different glasses and performed cataract surgery without result. The further progress of his visual symptoms was dismissed as an ocular cause and ignored by family.

Since patients can often express themselves very coherently, they are not considered as having a “dementia” which most likely happened with our patient as well. While early stages of Alzheimer's disease often manifest with comparatively maintained episodic memory, executive functioning, language, and insight, increasing neurodegeneration ultimately results in greater generalised damage. This means that by the time patients appear to neurologists or psychiatrists, PCA is typically in a very advanced stage. Also like many other patients, our patient was diagnosed late, about six years after the onset of his visual symptoms. The early visual features of PCA like simultanagnosia, optic ataxia and occulomotor apraxia are difficult to analyse. Simultanagnosia which is considered one of the early visual signs of PCA can probably be detected by the patient's inability to read the Ishihara pseudoisochromatic plates despite preserved color perception. Boston cookie-theft picture, and reading fragmented letters are other tests that can be further administered to confirm simultanagnosia in patients with isolated visual symptoms. Assessment of some of the early non-visual symptoms like acalculia and agraphia can also be done in a patient with vague visual complaints with a diagnostic suspicion of PCA, to aid in its early diagnosis. The onset of behavioural and psychological symptoms was probably the reason why his family had approached a psychiatrist. On cross sectional and longitudinal assessment of our patient's presentation, a differential diagnosis of delirium superimposed on dementia, atypical dementia (PCA), dementia with Lewy bodies and dementia in Parkinson's disease was considered. However on physical and neurological examination, laboratory investigations and imaging, we were able to narrow the diagnosis to posterior cortical atrophy, possibly Alzheimer’s disease. Commonly, people with PCA have trouble with tests, such as the "fragmented letters," "dot counting," and "A" cancellation tests [1]. Our patient struggled similarly with the ACE's disjointed letter and dot counting tasks. The functional repercussions of a visual impairment combined with dyspraxia are substantial. Most people with PCA lose their sight and become more reliant on others, which may cause feelings of helplessness, depression, anxiety, and guilt [9]. Likewise, our patient also had significant depressive symptoms that warranted treatment with antidepressants. Acetylcholinesterase inhibitors or memantine have been demonstrated to be helpful for people with PCA owing to Alzheimer’s disease [10]. The provision of practical and psychological support to affected individuals and their carers is the mainstay of management of patients with PCA (as with conventional typical Alzheimer’s dementia).

## Conclusions

In the early stages of PCA, visual symptoms predominate, while episodic memory, executive functioning, language, and insight are substantially retained. The time from first symptom to diagnosis is protracted, making it more likely that a PCA patient will first be diagnosed at a stage of significant functional impairment. Better identification, prognosis, and treatment of PCA will result from increased knowledge and understanding of the condition among neurologists, psychiatrists, general doctors, ophthalmologists, and optometrists.
